# Virulence of *Burkholderia pseudomallei* ATS2021 Unintentionally Imported to United States in Aromatherapy Spray

**DOI:** 10.3201/eid3010.240084

**Published:** 2024-10

**Authors:** Christopher K. Cote, Kevin D. Mlynek, Christopher P. Klimko, Sergei S. Biryukov, Sherry Mou, Melissa Hunter, Nathaniel O. Rill, Jennifer L. Dankmeyer, Jeremey A. Miller, Yuli Talyansky, Michael L. Davies, J. Matthew Meinig, Stephanie A. Halasohoris, Anette M. Gray, Jade L. Spencer, Ashley L. Babyak, M. Kelly Hourihan, Bobby J. Curry, Ronald G. Toothman, Sara I. Ruiz, Xiankun Zeng, Keersten M. Ricks, Tamara L. Clements, Christina E. Douglas, Suma Ravulapalli, Christopher P. Stefan, Charles J. Shoemaker, Mindy G. Elrod, Jay E. Gee, Zachary P. Weiner, Ju Qiu, Joel A. Bozue, Nancy A. Twenhafel, David DeShazer

**Affiliations:** United States Army Medical Research Institute of Infectious Diseases, Fort Detrick, Frederick, Maryland, USA (C.K. Cote, K.D. Mlynek, C.P. Klimko, S.S. Biryukov, S. Mou, M. Hunter, N.O. Rill, J.L. Dankmeyer, J.A. Miller, Y. Talyansky, M.L. Davies, J.M. Meinig, S.A. Halasohoris, A.M. Gray, J.L. Spencer, A.L. Babyak, M.K. Hourihan, B.J. Curry, R.G. Toothman, S.I. Ruiz, X. Zeng, K.M. Ricks, T.L. Clements, C.E. Douglas, S. Ravulapalli, C.P. Stefan, C.J. Shoemaker, J. Qiu, J.A. Bozue, N.A. Twenhafel, D. DeShazer);; Centers for Disease Control and Prevention, Atlanta, Georgia, USA (M.G. Elrod, J.E. Gee, Z.P. Weiner)

**Keywords:** *Burkholderia pseudomallei*, melioidosis, mice, ATS2021, Indian strain, biofilm, neurologic, bacteria, imported, United States

## Abstract

In the United States in 2021, an outbreak of 4 cases of *Burkholderia pseudomallei*, the etiologic agent of melioidosis and a Tier One Select Agent (potential for deliberate misuse and subsequent harm), resulted in 2 deaths. The causative strain, *B. pseudomallei* ATS2021, was unintentionally imported into the United States in an aromatherapy spray manufactured in India. We established that ATS2021 represents a virulent strain of *B. pseudomallei* capable of robust formation of biofilm at physiologic temperatures that may contribute to virulence. By using mouse melioidosis models, we determined median lethal dose estimates and analyzed the bacteriologic and histopathologic characteristics of the organism, particularly the potential neurologic pathogenesis that is probably associated with the *bimA_Bm_* allele identified in *B. pseudomallei* strain ATS2021. Our data, combined with previous case reports and the identification of endemic *B. pseudomallei *strains in Mississippi, support the concept that melioidosis is emerging in the United States.

In the United States, melioidosis is an emerging infectious disease caused by *Burkholderia pseudomallei* (*1*,*2*). In melioidosis-endemic areas, the bacterium causes pneumonia and fatal bacteremia with diverse mortality rates that depend on the standard of care provided, patient risk factors (e.g., diabetes), and extent and location of the infection (*3*,*4*). Melioidosis clinical manifestation ranges from asymptomatic to acute pulmonary severe illness or chronic infection (*1*). In a subset of patients (1.5%–5%), *B. pseudomallei* infection leads to serious neurologic melioidosis (*5*,*6*). Clinical signs/symptoms are fever, headache, seizures, unilateral weakness, paralysis, encephalomyelitis, and brain abscesses. Treatment duration is typically >6 months and includes an intravenous intensive phase and an oral eradication phase (*7*). Most cases of neurologic melioidosis are reported in the melioidosis-endemic areas of Australia and India (*8*–*10*).

In 2021, melioidosis was confirmed in 4 patients in the United States who had not traveled internationally (*11*). Whole-genome sequencing and epidemiologic investigations linked the clonal isolates of *B. pseudomallei* ATS2021 from all 4 patients to an aromatherapy spray imported from India. Neurologic melioidosis affected 2 of the patients; 1 died and 1 experienced long-term sequelae (*11*). Of the 2 cases of melioidosis that were not shown to have substantial neurologic involvement, 1 was fatal and the other patient recovered. In addition, a pet raccoon that had direct contact with the contaminated spray exhibited neurologic signs approximately 2 weeks after contact with the contaminated spray and 3 days before death. Although viable bacteria were not recovered from the carcass, tissue samples were positive by PCR for *B. pseudomall*ei DNA and provide support that the likely cause of death was neurologic melioidosis (*12*).

Inhaled *B. pseudomallei* can enter the central nervous system (CNS) through several portals (*13*–*15*). Previous studies in mice demonstrated colonization at the nasal mucosa-associated lymphoid tissue and the olfactory epithelium (*16*). Tracking *B. pseudomallei* after intranasal exposure demonstrates that the bacterium crosses the respiratory epithelium, but invasion likely requires that the olfactory epithelium be previously damaged (*16*). Neurologic disease is associated with the *Burkholderia* intracellular motility factor A (BimA) (*17*,*18*), a protein that is essential for actin-based motility, enabling intracellular movement and evasion of the immune system (*18*). Although all *B. pseudomallei* strains possess *bimA*, a subset encodes a variant gene (*bimA_Bm_*) that is 95% homologous to the gene in the closely related *Burkholderia mallei*, which correlates with neurologic involvement (*8*,*18*). In our study, we characterized *B. pseudomallei* ATS2021 and laid the groundwork for using that strain to test medical countermeasures against melioidosis.

## Materials and Methods

### Growth Curve Determinations

*B. pseudomallei* strains were grown in either Luria broth with 4% glycerol (LBG), 4% glycerol, 1% tryptone, 5% NaCl broth (GTB), M9 (minimal medium) + 2 % glucose, (M9G), or M9G + 0.5% casamino acids (M9GC). We resuspended strains from an overnight broth culture to a 600 nm optical density (OD_600_) of 0.5 in brain–heart infusion broth. We diluted suspensions 1:10 into a 96-well microtiter plate, grew them in a Spark (Tecan Group, https://www.tecan.com) microplate reader shaken at 37°C for ≈36 hours, and measured OD_600_ hourly. We obtained data by subtracting the value of the respective medium-only control from the measured OD_600_.

### Biofilm Assay

 We used crystal violet staining to measure biofilm. We resuspended *B. pseudomallei* overnight broth cultures to an OD_600_ of ≈0.2 in phosphate-buffered saline (PBS) and diluted the bacterial suspensions 1:10 into LBG, GTB, M9G, or M9GC in CoStar polystyrene 96-well plates, incubated at room temperature or 37°C for 24 or 48 hours. Before staining, we measured the OD_600_, after which we aspirated plates, washed 3 times with PBS to remove planktonic cells, and fixed with 100% ethanol for 30 minutes at room temperature. After fixing the samples in ethanol, we added 0.1% (wt/vol) crystal violet to each well for 15 minutes, and washed 3 times with PBS, after which we solubilized the remaining stain in 33% acetic acid. To quantify staining as an indicator of biofilm formation, we measured the OD_600_. When necessary, we diluted samples in 33% acetic acid to ensure that readings were within linear range. We averaged >3 technical replicates in each experiment. Data reported are the result of >4 individual experiments.

### Mouse Exposure to Aerosolized Bacteria

We started cultures by inoculating GTB with frozen bacterial stocks and growing them at 37°C while shaken at 200 rpm. We estimated bacterial concentrations by 620 nm OD (OD_620_) and then determined CFU by growing *B. pseudomallei* on sheep blood agar plates (Remel; Thermo Fisher Scientific, https://www.thermofisher.com). We exposed mice to aerosolized *B. pseudomallei* ATS2021 at increasing concentrations in a whole-body aerosol exposure chamber equipped with the automated Biaera particle generator (*19*). The small-particle aerosol size diameter is ≈1–3 μm. To estimate inhaled doses, we serially diluted samples collected by using an all-glass impinger onto sheep blood agar plates (*20*).

### Mouse 50% Lethal Dose Determinations and Bacterial Burden

At the time of exposure to aerosolized *B. pseudomallei*, female C57BL/6 and BALB/c mice (Charles River Laboratory) were 7–9 weeks of age. We exposed 48 C57BL/6 mice to 5 aerosolized doses of *B. pseudomallei* (Table). To calculate the 50% lethal dose (LD_50_), we observed a subset of those mice for clinical signs for 60 days after exposure. We serially euthanized other mice for histopathologic and bacteriologic analyses. We also determined LD_50_ for BALB/c mice.

**Table:** Median lethal dose of aerosolized *Burkholderia mallei* ATS2021 in C57BL/6 and BALB/c mice*

Mice†	Day	Inhaled dose	LD_50_ (95% CI)	TTD median (95% CI)	TTD mean (SE)
C57BL/6	21	9.9 × 10^–1^	56.4 CFU (21.3–150.2)	>21	>21
		1.32 × 10^1^		>21	16.5 (0.7)
		1.07 × 10^2^		18.0 (11.0–NC)	15.9 (0.9)
		1.15 × 10^3^		4.0 (3.0–5.0)	4.4 (0.3)
		4.49 × 10^3^		3.0 (NC)	3.1 (0.1)
	60	9.9 × 10^–1^	5.8 CFU (2.0–15.1)	>60	51.0 (NC)
		1.32 × 10^1^		41.5 (12.0– NC)	39.3 (5.6)
		1.07 × 10^2^		18.0 (11.0–26.0)	20.9 (3.2)
		1.15 × 10^3^		4.0 (3.0–5.0)	4.4 (0.3)
		4.49 × 10^3^		3.0 (NC)	3.1 (0.1)
BALB/c	21	4.00 × 10^–1^	4.1 CFU (1.8–11.3)	>21	4.0 (NC)
		2		>21	4.9 (0.1)
		2.05 × 10^1^		4.0 (3.0 –4.0)	4.3 (0.3)
		1.86 × 10^2^		3.0 (NC)	3.1 (0.1)
		9.60 × 10^2^		3.0 (2.0–3.0)	2.9 (0.1)
	60	4.00 × 10^–1^	4.1 CFU (1.8–11.3)	>60	4.0 (NC)
		2		>60	4.9 (0.1)
		2.05 × 10^1^		4.0 (3.0–4.0)	4.3 (0.3)
		1.86E × 10^2^		3.0 (NC)	3.1 (0.1)
		9.60 × 10^2^		3.0 (2.0–3.0)	2.9 (0.1)

*LD_50_, 50% lethal dose; NC, noncalculable; TTD, time to death or euthanasia in accordance with early intervention criteria. †Female; 7–9 weeks of age at time of exposure to aerosolized bacteria**.**

### Capsule Detection

We detected *B. pseudomallei* capsular polysaccharide (CPS) in brain homogenates by using an antigen capture immunoassay developed on the Magpix platform (Thermo Fisher Scientific). We coupled *Burkholderia* CPS antibody, 4C4 (*21*), to magnetic beads by using a Luminex xMap kit and biotinylated by using an EZ-link Sulfo-NHS-LC-Biotin kit (Thermo Fisher Scientific). We added 4C4-coupled beads (2,500 beads/well), and 50 μL of study samples diluted 1:20 in 5% skim milk in PBS with 0.5% Tween-20 (mixture called SM) to white, round-bottom 96-well plates and incubated for 1 hour with shaking. We washed the wells 3 times with 100 μL of PBS-T (PBS with Tween 20) before adding 50 μL of 4 μg/mL of biotinylated 4C4 in SM. After incubating the wells for 1 hour, we washed them before adding 50 μL of 10 μg/mL streptavidin phycoerythrin in SM. After the wells were incubated for 30 minutes, we washed the beads, resuspended then in 100 μL of PBS-T, and read them by using the Magpix instrument. We considered samples with a median fluorescent intensity 2-fold over naive brain homogenate to be positive.

### Histopathologic Analyses

 We examined a subset of mice histopathologically (Appendix 1 Table 1). We performed necropsies and processed the tissues after they had been in 10% buffered formalin for 21 days. We produced tissue blocks and slides and stained them with hematoxylin and eosin. We performed immunohistochemistry (IHC) on select animals by using the BOND RX Automated Stainer (Leica Biosystems, https://www.leicabiosystems.com). We used a rabbit polyclonal *Burkholderia* antibody (antibody no. 351; US Army Medical Research Institute of Infectious Diseases) at a dilution of 1:5,000. We counterstained the sections with hematoxylin and applied coverslips.

### RNA Extraction and Nanostring Data Collection

We inactivated all brain homogenates with a 3:1 ratio of TRIzol LS (Thermo Fisher Scientific) and extracted total RNA as previously reported (*22*). We collected host gene expression data by using the nCounter mouse Neuroinflammation Panel on the SPRINT Profiler platform (NanoString Inc, https://nanostring.com). The panel consists of 770 genes spanning 23 neuroinflammation pathways and processes. In brief, we added 70 μL of hybridization buffer to the reporter code set to make a master mixture. We added 8 μL of the master mixture to 50 ng of extracted host RNA and 2 μL of the capture code set, incubated it at 65°C for 17 hours, and then incubated at 4°C until the samples were placed on a NanoString SPRINT Profiler and total fluorescent counts corresponding to target binding were collected. We extracted gene expression analysis and cell profiling data from Nanostring RCC files and analyzed by using the ROSALIND Bioinformatics suite (https://rosalind.bio). We generated normalized counts by using criteria provided by NanoString. ROSALIND follows the nCounter Advanced Analysis protocol (NanoString) of dividing counts within a lane by the geometric mean of the normalizer probes from the same lane. Housekeeping probes to be used for normalization are selected based on the geNorm algorithm as implemented in the NormqPCR R library1 (*23*). Unless otherwise noted, we based significance of differentially expressed genes on them passing a filter greater than ±1.5-fold linear threshold and an adjusted p<0.05 relative to brain homogenates from an unchallenged mouse group. We based cell profiling data for oligodendrocytes on abundance scores provided by the ROSALIND NanoString Cell Type Profiling Module. We performed statistical analyses and graphing for host gene expression on Graphpad Prism 9.4.0 (https://www.graphpad.com).

### Statistical Analyses

We analyzed biofilm data analyzed by using a linear mixed effects model implemented in the GLIMMIX procedure of SAS version 9.4 (SAS Institute Inc., https://www.sas.com). We did not apply multiplicity adjustment. We estimated LD_50_ under a probit model with log_10_ transformation of the dose variable and obtained LD_50_ pairwise comparisons by using nonlinear mixed model on the log_10_ dose scale. We estimated median time to death or euthanasia in accordance with early endpoint euthanasia criteria and accompanying confidence limits, mean time to death, and SE by using Kaplan-Meier survival methods. We determined correlation between in vitro biofilm production and in vivo virulence by using Spearman rank order analyses. We performed analysis in SAS version 9.4.

## Results

### Genetic Analyses and Molecular Virulence Determinants of ATS2021 

**Figure 1:**
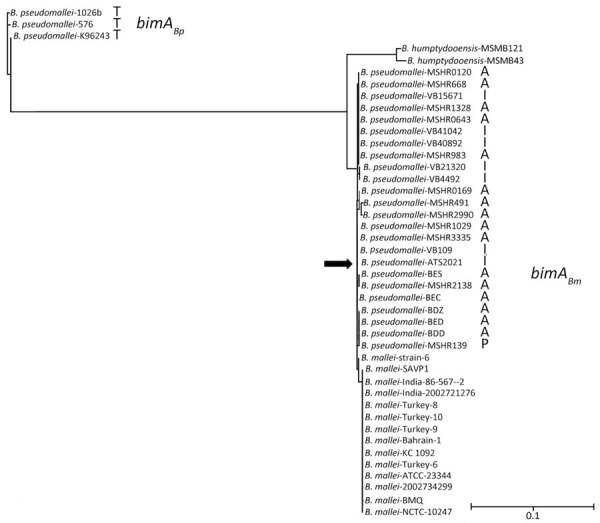
Phylogenetic tree based on *bimA_Bp_* and *bimA_Bm_* alleles from strains of *Burkholderia pseudomallei*, *B. mallei*, and *B. humptydooensis*, showing location of *B. mallei* ATS2021 (arrow), the causative strain in an outbreak of 4 cases, 2 of them fatal, in the United States in 2021. NGPhylogeny.fr (*12*) was used to build the tree in “A la Carte” mode, and it used MUSCLE for multiple alignment, Gblocks for automatic alignment curation (https://NGphylogeny.fr for both), PhyML-SMS (https://www.atgc-montpellier.fr) for tree inference, and exported to the Interactive Tree Of Life (iTOL; https://itol.embl.de) for display and manipulation. *B. pseudomallei* strains were isolated in Thailand (T), Australia (A), India (I), and Papua New Guinea (P). Scale bar indicates number of substitutions per site. *bimA*, *Burkholderia* intracellular motility factor A.

The ATS2021 whole-genome shotgun sequencing project was available in GenBank (accession no. JASCQT000000000.1). We examined the genetic sequence of known *B. pseudomallei* surface-associated virulence determinants, including the 6-deoxyheptan CPS (*24*), the lipopolysaccharide O-antigen (*25*), and the cluster 1 type 6 secretion system (*26*). Those gene clusters exhibited ≈99% identity with the corresponding gene clusters in the prototypic strain *B. pseudomallei* K96243 (*27*). In addition, immunoblot analysis with specific antibodies demonstrated the production of CPS and type A lipopolysaccharide O-antigen when ATS2021 was grown in vitro (data not shown). Petras et al. recently revealed that ATS2021 harbors a *bimA* gene that closely resembles the *bimA* gene found in *B. mallei*, the etiologic agent of glanders (*12*). The *bimA* gene encodes a trimeric autotransported protein that mediates actin-based motility in infected host cells (*28*). Most *B. pseudomallei* strains from environmental or clinical sources contain the *bimA_Bp_* allele, but a relatively small number of strains possess the *bimA_Bm_* allele and are often isolated from patients with neurologic melioidosis (*17*,*18*,*29*,*30*). The presence of the *bimA_Bm_* allele is thought to exacerbate neurologic melioidosis by increasing rapidity of bacterial dissemination to various tissues and persistence in phagocytic cells (*29*). The phylogenetic tree is based on the unique *bimA_Bm_* alleles deposited in publicly available draft and complete genome sequence databases (Figure 1). The *B. pseudomallei bimA_Bm_* sequences are all derived from strains originating in Australia, Papua New Guinea, and India, differing from each primarily in the region encoding the proline-rich domain (*28*,*30*). The ATS2021 *bimA* (Figure 1, arrow) groups within the *bimA_Bm_* branch of the tree. *B. pseudomallei* strains with the *bimA_Bm_* allele have also been described in Sri Lanka (*31*), but the sequences of those isolates have not been made publicly available. Of note, 2 environmental isolates of *Burkholderia humptydooensis*, MSMB 43 and MSMB121, harbor genes that are closely related to the *bimA_Bm_* alleles in *B. mallei* and *B. pseudomallei* (*32*) (Figure 1). To demonstrate the sequence differences between the *bimA_Bp_* and *bimA_Bm_* alleles, we included 3 representative *bimA_Bp_* alleles from K96243, 1026b, and 576 (Figure 1).

### ATS2021 Robust Biofilm 

**Figure 2:**
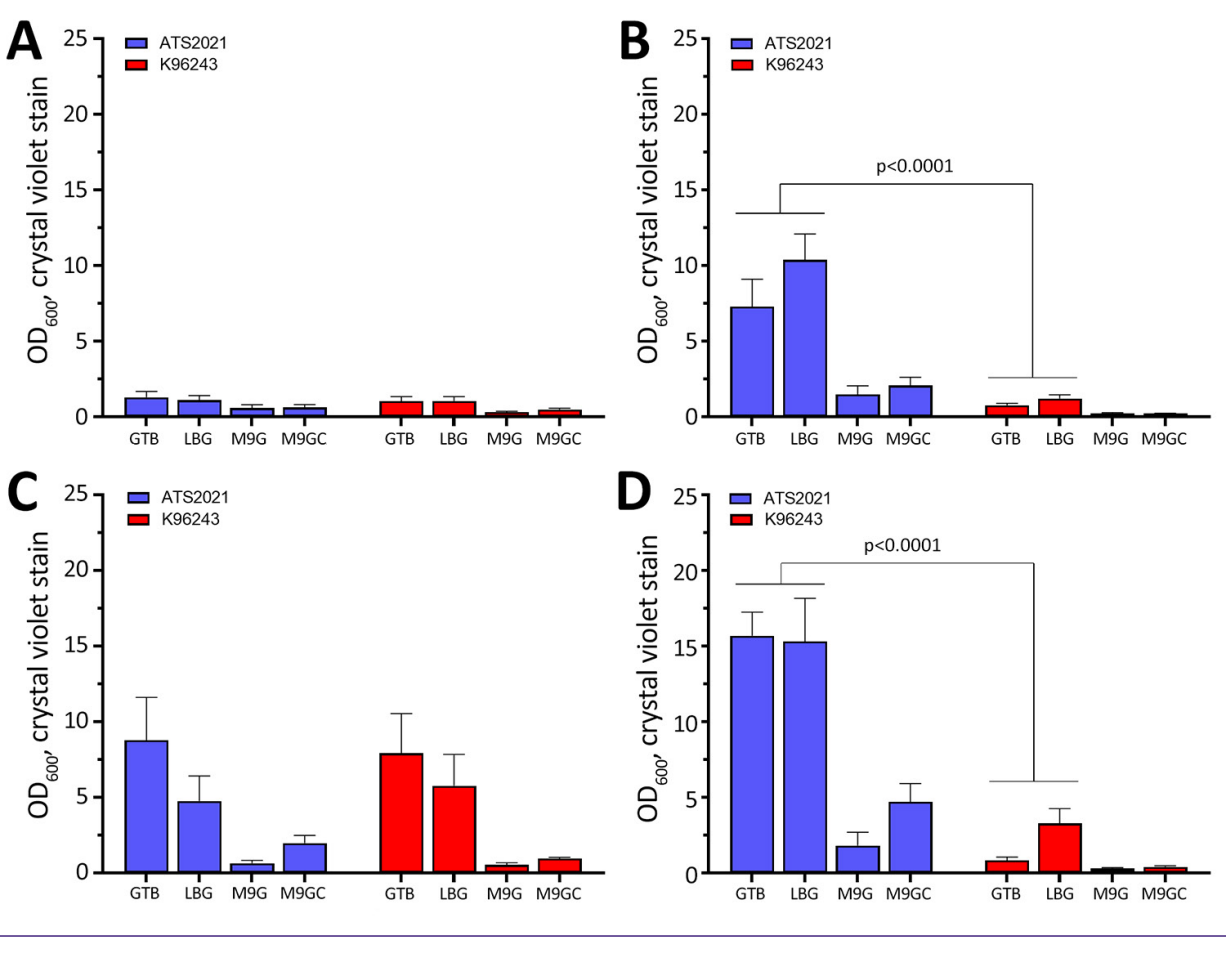
Biofilm formation of *Burkholderia pseudomallei* strains ATS2021, the causative strain in an outbreak of 4 cases, 2 of them fatal, in the United States in 2021, and K96243 under multiple test conditions. Biofilm formation of both strains was assessed by crystal violet staining as measured by OD_600_. A) One day at room temperature; B) 1 day at 37°C; C) 2 days at room temperature; D) 2 days at 37°C. Error bars represent the SEs from mean values determined from 3 separate assays. p values determined by linear mixed effects model. M9G, M9 + glucose; M9GC, 0.2% glucose to M9 and casamino acids; OD_600_, optical density at 600 nm.

**Figure 3:**
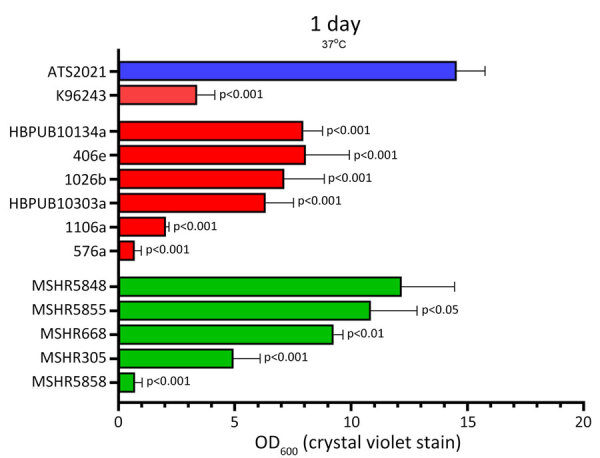
Biofilm production for *Burkholderia pseudomallei* strain ATS2021, the causative strain in an outbreak of 4 cases, 2 of them fatal, in the United States in 2021, in relation to other previously characterized *B. pseudomallei* clinical isolates. Biofilm formation of bacterial strains was assessed by crystal violet staining as measured by OD_600_. Biofilm was allowed to form after static growth in LB+4% glycerol for 1 day at 37°C. Clinical isolates used in this assay originated from Thailand (red bars) or Australia (green bars). Error bars represent the SEs from mean values determined from 4 independent assays. ATS2021 formed significantly more biofilm under these conditions compared to all isolates except MSHR5848 as determined by a linear mixed effects model. OD_600_, optical density at 600 nm.

We compared growth of K96243 and ATS2021 in several types of rich or defined media to assess differences with nutritional requirements and observed no differences in growth between the 2 strains (Appendix 2 Figure). We measured the ability of K96243 and ATS2021 to produce biofilm by using different growth media, incubation times, and temperatures. Low-level biofilm was detected for both strains after 1 day at room temperature (Figure 2). However, after 2 days at room temperature, levels of biofilm were higher for K96243 and ATS2021, especially in the rich media (LBG and GTB). The difference in biofilm formation was greater after incubation at 37°C; ATS2021 produced more biofilm than K96243. To determine if the increased formation at 37°C was specific to ATS2021, we tested biofilm formation in *B. pseudomallei* strains that accounted for the previously described strain panel (*33*). The clinical isolates formed variable biofilms, but ATS2021 produced substantially more biofilm at 37°C than the other strains, except MSHR5848 (Figure 3). Those differences were not observed when the bacteria were grown at room temperature (Figure 2; data not shown for all bacteria). Retrospective analyses demonstrated a negative correlation between in vitro biofilm formation and in vivo virulence in mice after exposure to aerosolized *B. pseudomallei* (Spearman correlation −0.70 and p = 0.017 for BALB/c mice; Spearman correlation −0.63 and p = 0.038 for C57BL/6 mice). Those analyses did not reveal statistically significant correlations between in vitro biofilm formation and in vivo virulence in mice after intraperitoneal injection of *B. pseudomallei*.

### LD_50_ Determinations in Mice

**Figure 4:**
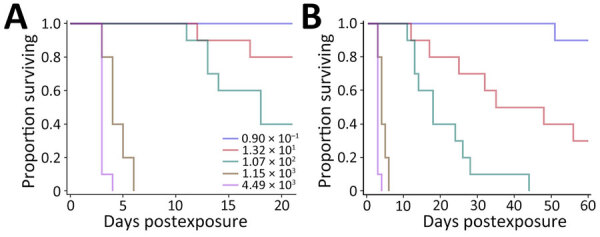
Kaplan-Meier survival plots calculated for C57BL/6 mice exposed to aerosolized *Burkholderia pseudomallei* ATS2021, the causative strain in an outbreak of 4 cases, 2 of them fatal, in the United States in 2021. A) Survival after 21 days; B) survival after 60 days. N = 10 per aerosolized dose of *B. pseudomallei.* The day 21 survival rates from highest to lowest calculated inhaled dose of *B. pseudomallei* ATS2021 (4,490 CFU, 1,150 CFU, 107 CFU, 13 CFU, 1 CFU) as depicted in panel A are 0/10, 0/10, 4/10, 8/10, and 10/10. The day 60 survival rates from highest to lowest calculated inhaled dose of *B. pseudomallei* ATS2021 as depicted in panel B are 0/10, 0/10, 0/10, 3/10, and 9/10.

**Figure 5:**
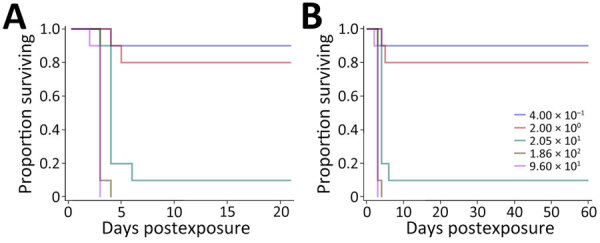
Kaplan-Meier survival plots calculated for BALB/c mice exposed to aerosolized *Burkholderia pseudomallei* ATS2021, the causative strain in an outbreak of 4 cases, 2 of them fatal, in the United States in 2021. A) Survival after 21 days; B) survival after 60 days. N = 10 per aerosolized dose of *B. pseudomallei.* The day 21 survival rates from highest to lowest calculated inhaled dose of *B. pseudomallei* ATS2021 (960 CFU, 186 CFU, 21 CFU, 2 CFU, 0.4 CFU) as depicted in panel A are 0/10, 0/10, 1/10, 8/10, and 9/10. The day 60 survival rates as depicted in panel B are 0/10, 0/10, 1/10, 8/10, and 9/10.

We used C57BL/6 mice because they are an accepted model for vaccine development (*34**,**35*). The LD_50_ estimate after exposure to aerosolized ATS2021 was ≈56 CFU after 21 days and 6 CFU after 60 days (Table; Figure 4). We also determined the LD_50_ in BALB/c mice because they are used for pathogenesis and therapeutic studies (*19*,*33*,*36*). The LD_50_ estimate for 21 and 60 days after exposure to aerosolized ATS2021 was ≈4 CFU for both time frames (Table; Figure 5). Those LD_50_ values categorize the isolate to be among the most virulent we have characterized. Retrospective statistical analyses supported this observation and demonstrated similar (p>0.1) virulence to *B. pseudomallei* strains 1026b, MSHR5855, HBPUB10303a, and HBPBUB10134a in C57BL/6 mice exposed to aerosolized bacteria (*19*). Clinical signs assumed to be associated with neurologic melioidosis were observed in some mice (e.g., uncoordinated and impaired movement, tremors, and hypersensitivity to touch).

### Bacterial Burden in C57BL/6 Mice after Exposure to Aerosolized ATS2021

**Figure 6:**
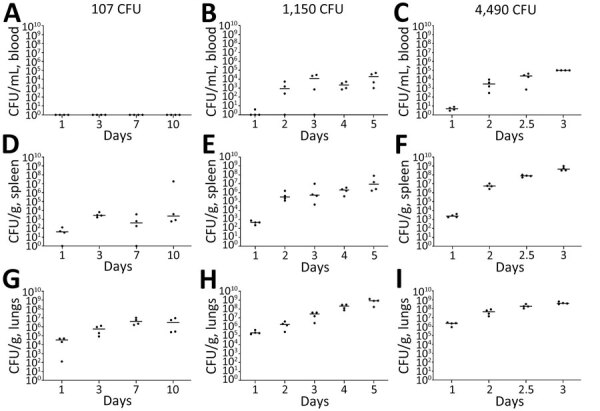
Serial sampling experiment to investigate bacterial dissemination in blood, spleens, and lungs of C57BL/6 mice at different challenge doses of aerosolized *Burkholderia pseudomallei* strain ATS2021, the causative strain in an outbreak of 4 cases, 2 of them fatal, in the United States in 2021. C57BL/6 mice were estimated to have inhaled various doses on day 0. A subset of mice was then deeply anesthetized for a terminal blood collection, euthanized, and then the spleens and lungs were removed to determine the bacteriologic burden in each organ at each time point indicated. Bacterial counts are shown for whole blood (A–C), spleen homogenate (D–F), and lung homogenate (G–I). N = 4 for each time point. The CFU burden is shown for each mouse; the geometric mean is depicted with the horizontal bar. Limit of detection is ≈100 CFU/mL of blood and 5 CFU/organ.

**Figure 7:**
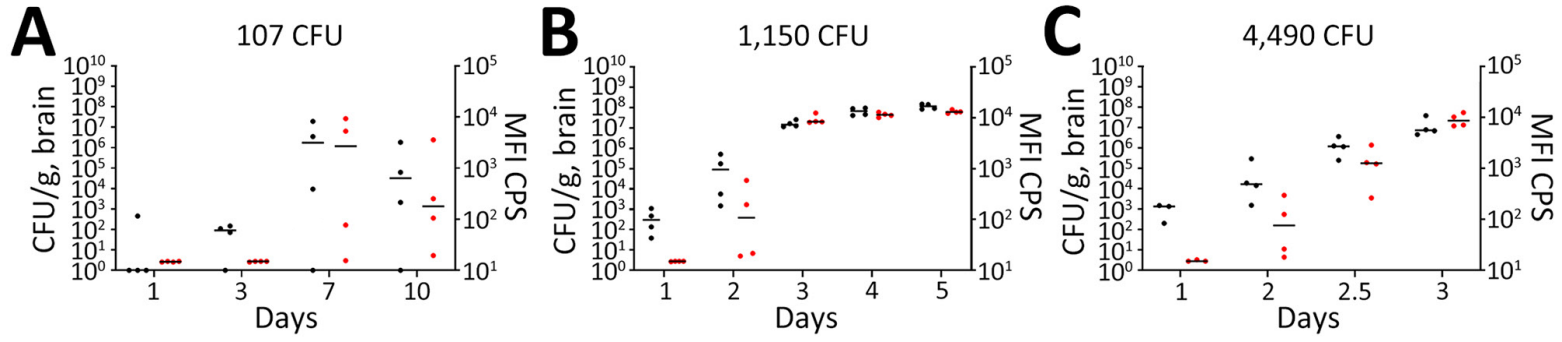
Serial sampling experiment to investigate bacterial dissemination in brains of C57BL/6 mice at different challenge doses of aerosolized *Burkholderia pseudomallei* strain ATS2021, the causative strain in an outbreak of 4 cases, 2 of them fatal, in the United States in 2021. C57BL/6 mice were estimated to have inhaled various doses of aerosolized *Burkholderia pseudomallei* ATS2021 on day 0: A) 107 CFU, B) 1,150 CFU, and C) 4,490 CFU. A subset of mice was then deeply anesthetized for a terminal blood collection, euthanized, and then brains were removed to determine the bacteriologic burden in each brain at each time point indicated. N = 4 for each time point. The CFU burden is shown for each mouse, depicted as a black circle; geometric mean is depicted with the horizontal bar. The brain homogenates were exposed to ≈21 kGy of gamma radiation, proven sterile, and then subjected to the *B. pseudomallei* capsule-specific immunodiagnostic assay. The MFI of each mouse is depicted by a red circle, and the geometric mean is depicted with the horizontal bar. Limit of detection is ≈5 CFU/organ. The detection of capsule in the irradiated brain homogenate is depicted in red and is displayed as MFI. MFI, mean fluorescent intensity.

We performed a serial sampling experiment to investigate bacterial dissemination in C57BL/6 mice at different challenge doses (107 CFU, 1,150 CFU, and 4,490 CFU). Bacterial dissemination patterns depend on the dose of inhaled bacteria after aerosolization (Figure 6). The 2 highest inhaled doses resulted in early bacteremia (Figure 6, panels B, C). *B. pseudomallei* was detected in the spleens (Figure 6, panels D–F) of nearly all animals within 24 hours. Regardless of the exposure dose, all mice had substantial bacterial replication in the lungs within 24 hours, as expected, given that the lungs were the main portal of entry for the bacteria (Figure 6, panels G–I). We examined the brains and identified substantial bacterial burden within 24 hours after inhalation in the 3 doses (Figure 7, black data points). We also performed a capsule-specific immune-diagnostic assay on gamma-irradiated brain homogenates. Detection of capsule in those samples (Figure 7, red data points) was reflective of the bacterial burden, but quantification of viable bacteria via culture was more sensitive.

### Representative Histopathology in C57BL/6 Mice after Inhalation of ATS2021

We performed histopathologic analyses on a subset of animals. Animals that died 1–3 days after aerosol exposure probably did not have time for substantial tissue lesions to develop (despite high exposure doses) and may have received lower severity scores because of rapid time to death or euthanasia, which accounts for the lowest animal scores at days 1, 2, and 3 in all 3 groups (Appendix 1 Table 1). Pathologic lesions in mice were consistent with lesions produced by other strains of *B. pseudomallei* (*19*,*37*). In general, the focal-to-coalescing necrotizing lesions were accompanied by many degenerate neutrophils and accumulating necrotic debris to form variably sized necrosuppurative lesions, which expand to form more organized abscesses with time.

**Figure 8:**
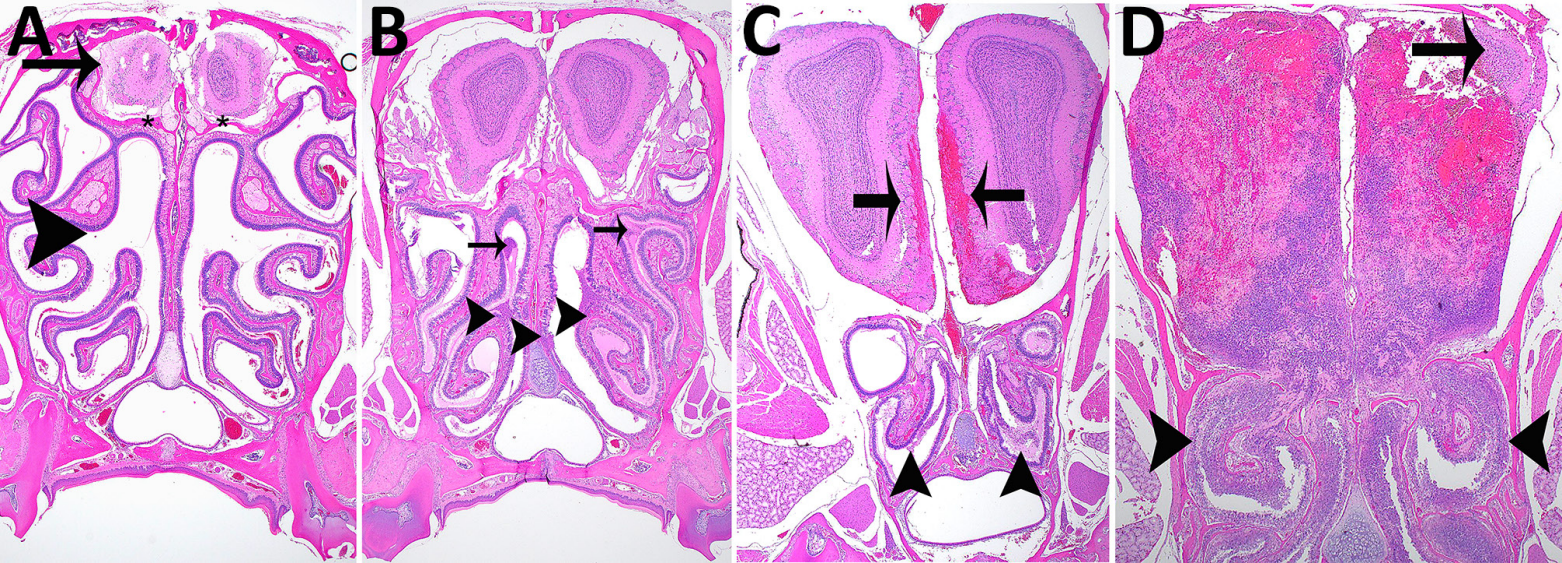
Hematoxylin and eosin staining of head sections of C57BL/6 mice exposed to aerosolized *Burkholderia pseudomallei* strain ATS2021, the causative strain in an outbreak of 4 cases, 2 of them fatal, in the United States in 2021. Shown are the nasal cavity (including nasal turbinates, nasal septum, respiratory and olfactory epithelium, lamina propria with supporting tissues and glands, nerve bundles, and nasal air passages); cribriform plate (bone and olfactory/trigeminal nerves); and cranial vault with olfactory bulb. A) Day 1 after exposure, dose 107 CFU, showing nasal turbinates (arrowhead), cribriform plate (asterisks), and olfactory bulb (arrow) that are essentially normal. Original magnification ×2. B) Day 2 after exposure, dose 4,490 CFU, showing mild necrosuppurative rhinitis (arrowheads) with edema, cellular debris and suppurative inflammation in few nasal air passages (arrows). Original magnification ×2. C) Day 3 after exposure, dose 4,490 CFU, showing multifocal moderate necrosuppurative rhinitis (arrowheads) and necrotizing and hemorrhagic meningoencephalitis of the olfactory bulb (arrows). Original magnification 2. D) Day 4 after exposure, dose 1,150 CFU. There is diffuse necrosuppurative rhinitis of the nasal turbinates (arrowheads), showing extensive necrosis and hemorrhage of the olfactory bulb with a small portion of recognizable neural tissue evident (arrow). Original magnification ×2.

**Figure 9:**
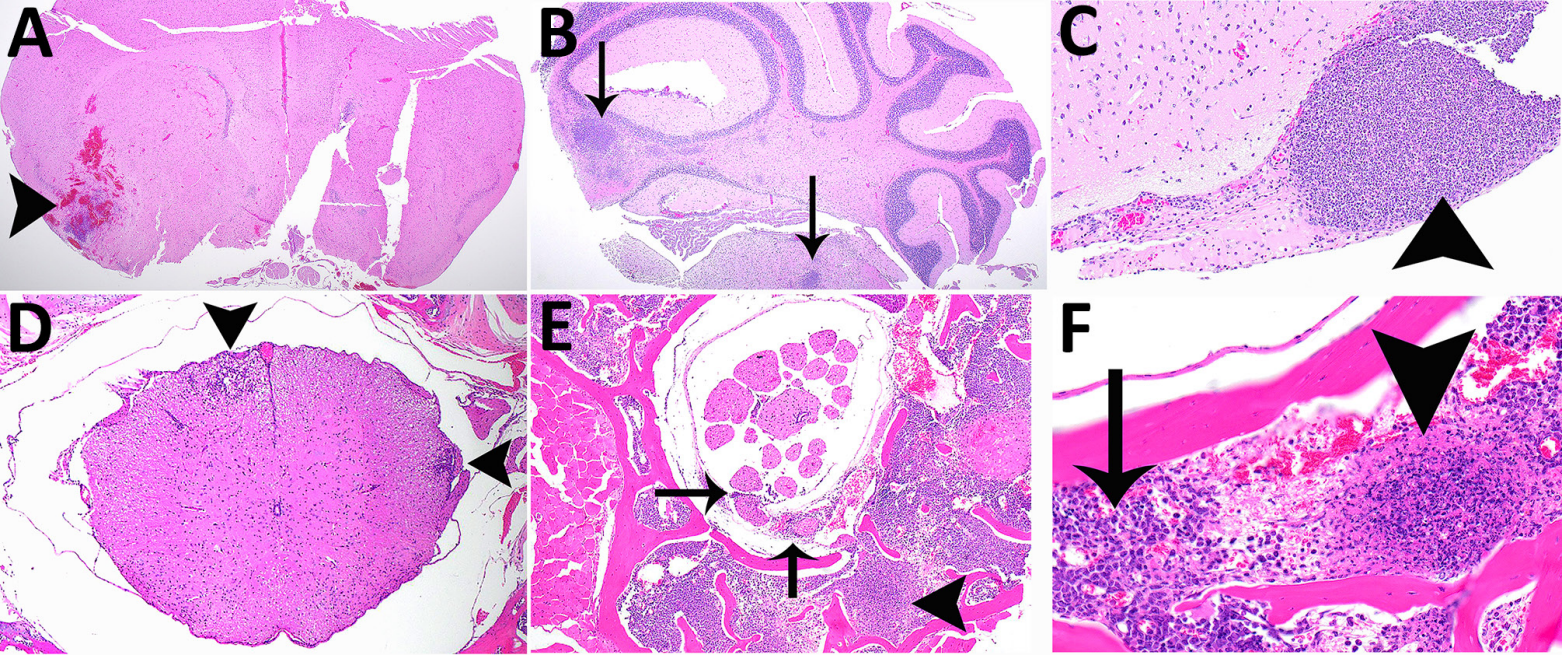
Histopathologic analyses of the neurologic system of C57BL/6 mice after inhalation of *Burkholderia pseudomallei* strain ATS2021, the causative strain in an outbreak of 4 cases, 2 of them fatal, in the United States in 2021. A) Day 5 after exposure, dose 1,150 CFU. Cerebrum showing focally extensive necrotizing and hemorrhagic meningoencephalitis (arrowhead). Hematoxylin and eosin (HE) stain; original magnification ×2. B) Day 9 after exposure, dose 107 CFU. Pons and cerebellum. There is multifocal necrotizing meningoencephalitis (arrows). HE stain; original magnification ×2. C) Day 10 after exposure, dose 1,150 CFU. Cerebrum with olfactory peduncle filled with viable and degenerate neutrophils with no recognizable peduncular tissue (arrowhead). HE stain; original magnification ×20. D) Day 9 after exposure, 107 CFU. Spinal cord, thoracic, shows multifocal meningomyelitis (arrowheads). HE stain; original magnification ×4. E) Day 5 after exposure, 1,150 CFU. Spinal cord and vertebrae, lumbar at cauda equina, show is multifocal suppurative perineuritis of spinal nerves (arrows). Note the necrotizing lesion within the vertebral bone marrow (arrowhead) HE stain; original magnification ×10. F) Day 4 after exposure, dose 1,150 CFU. Spinal cord and vertebra, thoracic, show necrotizing osteomyelitis (arrowhead). Note the loss of distinction of bone marrow cells compared to normal cells of the bone marrow (arrow). HE stain; original magnification ×40.

We prioritized the central nervous system to characterize the ATS2021 isolate after inhalation of small-particle aerosols (Figures 8,9). Lesions consistent with *B. pseudomallei* were seen by day 1 in the lung and nasal turbinates, the sites of initial colonization. Subsequently, lesions were noted by day 2 in the brain, specifically the olfactory bulb, and in the olfactory nerves of many mice from 1,150 and 4,490 CFU doses. In addition, by day 2, lesions were present in the spleen and liver, as noted by increased pathology scores (Appendix 1 Table 1). Lesions of the vertebral bone marrow were noted after 1,150 and 4,490 CFU doses within 3 days. Bone marrow lesions were primarily noted in the groups that received higher doses. Lesions of the cerebrum, cerebellum, and brainstem were noted by day 4 and spinal cord lesions by day 5 (Figure 9). Lesions were noted in the olfactory bulb in mice that received doses of 1,150 CFU and in the olfactory nerves of mice that received 1,150 CFU and 4,490 CFU within 48 hours after exposure and an increased number of mice with lesions in the olfactory bulb by 36 hours.

**Figure 10:**
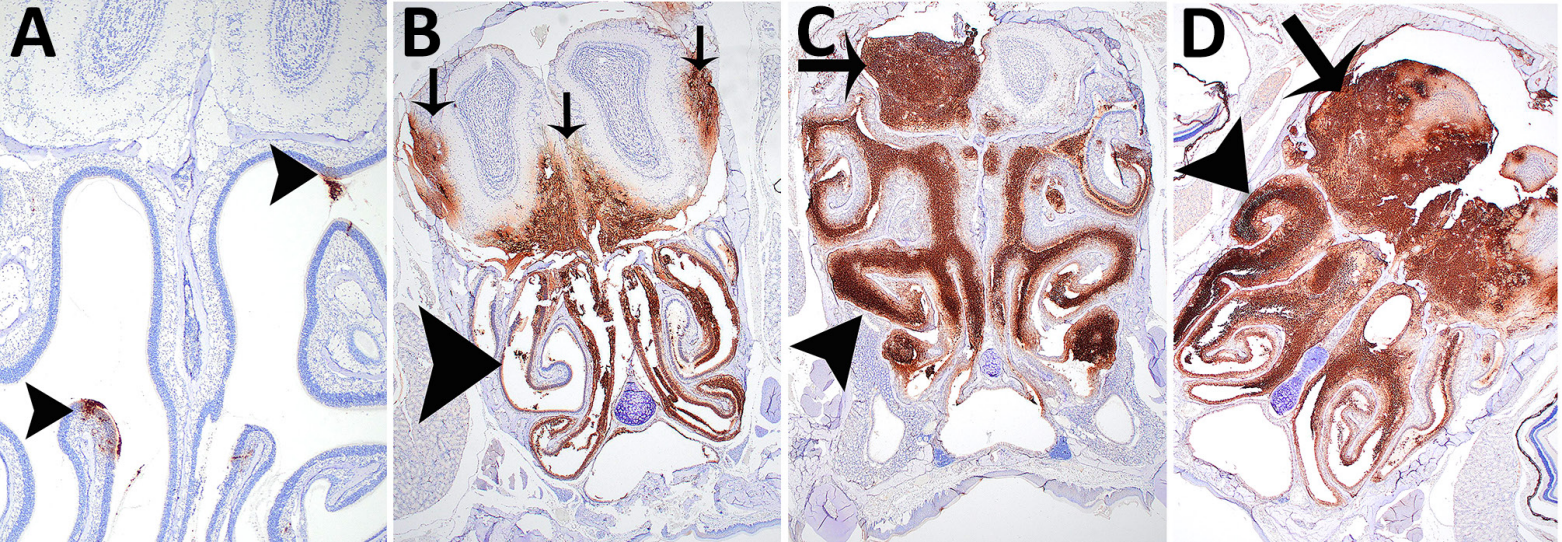
Immunohistochemical analyses of head sections of C57BL/6 mice exposed to aerosolized *Burkholderia pseudomallei* strain ATS2021, the causative strain in an outbreak of 4 cases, 2 of them fatal, in the United States in 2021. Shown are the nasal cavity (including nasal turbinates, nasal septum, respiratory and olfactory epithelium, lamina propria with supporting tissues and glands, nerve bundles, and nasal air passages); cribriform plate (bone and olfactory/trigeminal nerves); and cranial vault with olfactory bulb. A) Day 1 after exposure, dose 1,150 CFU, shows multifocal minimal positivity of the nasal cavity epithelium (arrowheads), which is most likely olfactory epithelium of the ethmoid turbinate. Original magnification ×2. B) Day 3 after exposure, dose 4,490 CFU, showing diffuse marked positivity of the nasal cavity epithelium (arrowhead) and multifocal moderate positivity of the olfactory bulb (arrows). Original magnification ×2. C) Day 6 after exposure, dose 107 CFU, showing diffuse severe positivity of the nasal cavity epithelium (arrowhead) and multifocal marked positivity of the olfactory bulb (arrow). Original magnification ×2. D) Day 4 after exposure, 1,150 CFU, showing diffuse severe positivity of the nasal cavity (arrowhead) and olfactory bulb (arrow). Originalmagnification ×2.

**Figure 11:**
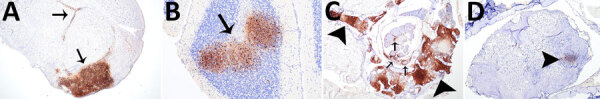
Immunohistochemical analyses of the neurologic system of C57BL/6 mice exposed to aerosolized *Burkholderia pseudomallei* strain ATS2021, the causative strain in an outbreak of 4 cases, 2 of them fatal, in the United States in 2021. A) Day 6 after exposure, dose 107 CFU. Cerebrum shows multifocal moderate positivity (arrows). Original magnification ×2. B) Day 6 after exposure, dose 107 CFU. Cerebellum shows multifocal moderate positivity (arrow). Original magnification ×10. C) Day 5 after exposure, dose 1,150 CFU. Lumbar spinal cord at cauda equina and vertebra shows multifocal mild perineural positivity (arrows) of spinal nerves at cauda equina and marked vertebral bone marrow positivity (arrowheads). Original magnification ×10. D) Day 9 after exposure, dose 107 CFU. Spinal cord, cervical, shows focal moderate positivity (arrowheads). Original magnification ×20.

We performed IHC to support histopathologic findings and provide additional evidence that the lesions resulted from infection with *B. pseudomallei* (Appendix 1 Table 2). IHC clearly identified bone marrow lesions that are difficult to appreciate with hematoxylin and eosin staining. Nasal turbinates showed *Burkholderia* positivity by IHC within 24 hours after exposure, and IHC positivity was found within 48 hours in the mice receiving the highest inhaled dose in the brain olfactory bulb, olfactory nerves, and nasal turbinates (Figures 10,11).

### Effects on Oligodendrocytes

**Figure 12:**
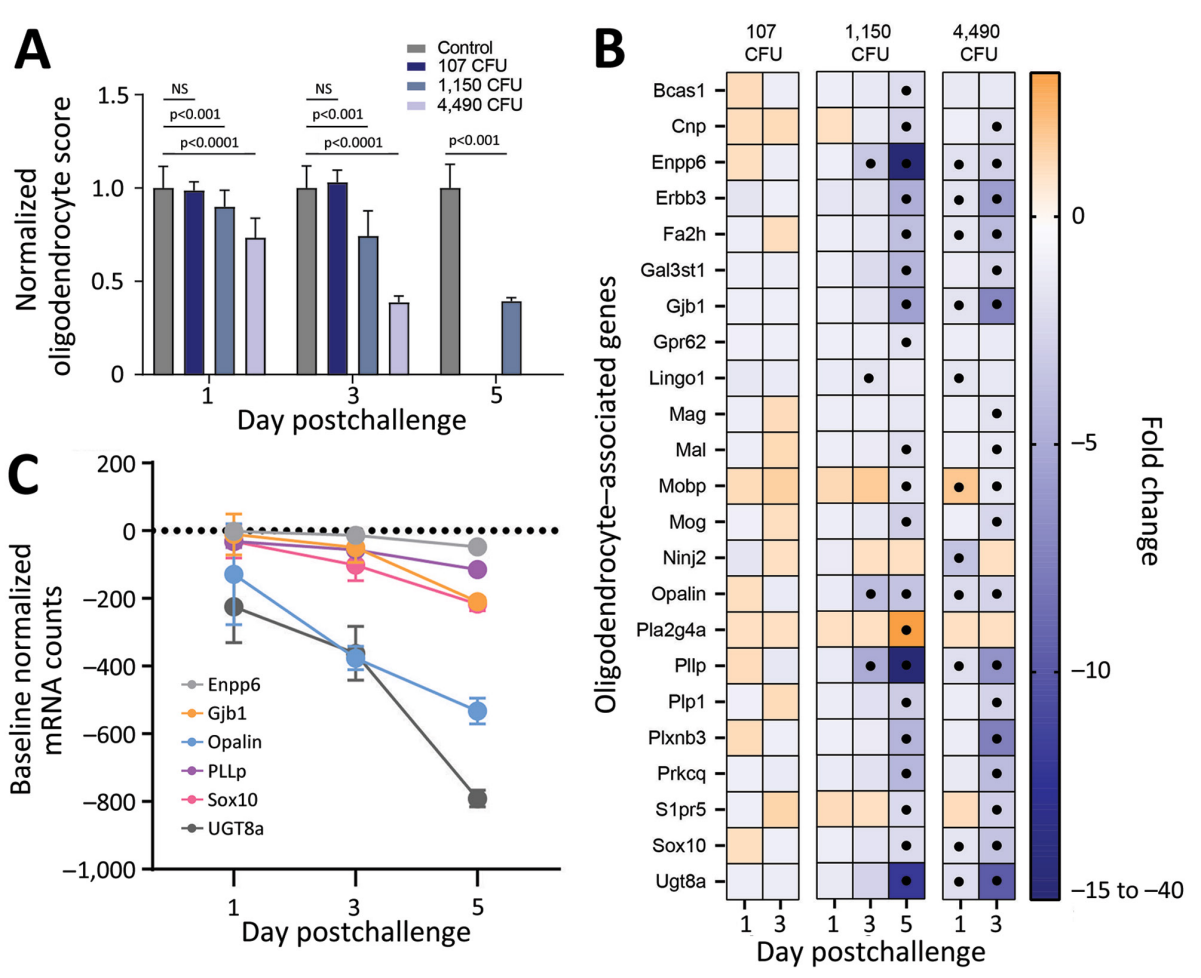
Profiling of differentially expressed genes revealing downregulation of markers associated with oligodendrocytes after exposure to aerosolized *Burkholderia pseudomallei* strain ATS2021, the causative strain in an outbreak of 4 cases, 2 of them fatal, in the United States in 2021. A) Significant downregulation or decreased expression of genes involved in oligodendrocyte function was observed in select challenge groups of mice on days 1,3, and 5 after challenge. Values are normalized to unchallenged control mice and represent 4 mice per group. Significance based on a 2-way analysis of variance. Error bars indicate 95% CIs. B) Heat map of differentially expressed genes associated with NanoString oligodendrocyte cell profiler panel (https://nanostring.com). Fold change is linear, and dots indicate gene changes that were above the significance threshold. C) Expression changes over time in select genes associated with oligodendrocyte function for the 1,150 CFU challenge group. In panels A and C, error bars represent standard deviation.

We applied a transcriptomic approach targeted against a range of neuroinflammatory genes in brain homogenates from mice. According to targeted expression levels, we used a cell profiling module to identify changes in neuronal cell types based on relative abundance of marker genes associated with oligodendrocytes. In the 2 highest dose groups, we observed statistically significant drops in oligodendrocyte signatures, particularly on days 3 and 5 (Figure 12, panel A). A total of 27 genes constitute the oligodendrocyte cell profile, of which 23 had significant expression differences for >1 time point (Figure 12, panel B). Total numbers of significant genes were dependent on infectious dose with no differential gene expression observed in the 107 CFU group. We observed early and pronounced down-regulation of many of these genes, as early as day 1 in the 4,490 CFU group. Of the 27 genes, 6 are known to predominately constitute the oligodendrocyte cell profile and all encode proteins involved in myelination or lipid metabolism (Appendix 2 Table). All 6 of the signatures were significantly down-regulated, further suggesting dysregulation in oligodendrocytes in infected hosts (Figure 12, panel C).

## Discussion

Knowledge of the diversity of virulence associated with distinct geographic isolates of *B. pseudomallei* is crucial to the further development, testing, and evaluation of medical countermeasures. We characterized several factors that may contribute to the virulence associated with ATS2021. We examined several key genetic virulence attributes of that strain and demonstrated considerable virulence in 2 mouse models of inhalational melioidosis. Because ATS2021 was shown to carry the *bimA_Bm_* allele and neurologic involvement of this strain played a role in human infections, we focused on analyzing the histopathology associated with the rapid neurologic invasion. The nasal turbinates were heavily colonized within the first day after exposure to aerosolized bacteria, leading to infection of the olfactory bulb by day 2; the cerebrum, cerebellum, and brain stem by day 4; and the spinal column by day 5 (with evidence of infection in vertebral bone marrow within 3 days for mice inhaling the highest dose of aerosolized bacteria).

RNA analyses indicated that the oligodendrocyte populations in the infected mouse brains were significantly affected by the infection in a dose- and time-dependent manner. The robust decreases in oligodendrocyte abundance, function, or both that we observed in the highest bacterial challenge groups (Figure 12, panels A, B) suggest pronounced impairment of a glial cell population that is critical for brain homeostasis and function. Oligodendrocytes are responsible for myelination of the CNS, encapsulating neuronal axons in lipid-rich membranes that serve as insulators enabling rapid neuronal conduction (*38**,**39*). Given the role of oligodendrocytes in brain function, a variety of neurodegenerative disorders affecting the brain and spinal cord are associated with demyelination (e.g., multiple sclerosis) (*40*,*41*). There are known microbial-associated causes of oligodendrocyte dysfunction and associated demyelination; progressive multifocal leukoencephalopathy is caused by infection of oligodendrocytes by the JC virus (*42*), and *Borrelia burgdorferi* can induce oligodendrocyte apoptosis (*43*). Although more study is needed, the down-regulation of genes associated with myelination could account for some of the clinical signs observed in mice and nonhuman primates when infected with neurotropic strains of *B. pseudomallei*, including muscle weakness, tremors, and extreme sensitivity to touch (*37*,*44*).

Last, we are intrigued by the robust biofilm production at 37°C compared with other strains of *B. pseudomallei*. Although virulence (assayed by an intraperitoneal BALB/c infection model) did not correlate with biofilm production of *B. pseudomallei* isolates from Thailand (*45*), biofilm formation is associated with virulence of other bacteria (*46*,*47*). That this biofilm phenotype is exaggerated at 37°C suggests a potential role in pathogenesis that may be relevant to inhaled *B. pseudomallei* in the context of colonization of upper respiratory areas (e.g., nasal turbinates as the result of inhaling the bacteria) and may contribute to the intrinsic difficulty in successfully treating melioidosis with antimicrobial drugs (*48*). Our retrospective analyses support the data published by Taweechaisupapong et al., which demonstrate no correlation between biofilm formation and virulence associated with intraperitoneal injection (*45*), but we did identify a statistically significant negative correlation between in vitro biofilm formation and virulence as measured by mouse models of inhalational melioidosis. Future work focused on identifying correlations between biofilm formation and neurologic melioidosis should help clarify the effect of host–bacterial interactions on CNS demyelination.

Combined, our data, previous case-reports (*11*,*12*), and the identification of endemic strains in Mississippi (*49*) support the idea that melioidosis is an emerging infectious disease in the United States. Thus, it is imperative that we understand the new isolates in the context of inhalational and neurologic melioidosis to accurately predict the hazards associated with this emerging pathogen for the biodefense and public health communities. Clinical laboratories must continue to be on the alert for melioidosis within the United States, and new isolates should be used to develop and test novel medical countermeasures and diagnostic strategies.

Appendix 1 Details of results from mouse testing in study of virulence of *Burkholderia pseudomallei* ATS2021 unintentionally imported to united states in aromatherapy spray.

Appendix 2 Additional information for study of virulence of *Burkholderia pseudomallei* ATS2021 unintentionally imported to united states in aromatherapy spray.

## References

[1] Currie BJ. Melioidosis: evolving concepts in epidemiology, pathogenesis, and treatment. Semin Respir Crit Care Med. 2015;36:111–25. 10.1055/s-0034-1398389 25643275

[2] Currie BJ, Meumann EM, Kaestli M. The expanding global footprint of *Burkholderia pseudomallei* and melioidosis. Am J Trop Med Hyg. 2023;108:1081–3. 10.4269/ajtmh.23-0223 37160279 PMC10540122

[3] Chantratita N, Phunpang R, Yarasai A, Dulsuk A, Yimthin T, Onofrey LA, et al. Characteristics and one year outcomes of melioidosis patients in northeastern Thailand: a prospective, multicenter cohort study. Lancet Reg Health Southeast Asia. 2023;9:9. 10.1016/j.lansea.2022.100118 36570973 PMC9788505

[4] Currie BJ, Mayo M, Ward LM, Kaestli M, Meumann EM, Webb JR, et al. The Darwin Prospective Melioidosis Study: a 30-year prospective, observational investigation. Lancet Infect Dis. 2021;21:1737–46. 10.1016/S1473-3099(21)00022-0 34303419

[5] Currie BJ, Fisher DA, Howard DM, Burrow JN. Neurological melioidosis. Acta Trop. 2000;74:145–51. 10.1016/S0001-706X(99)00064-9 10674643

[6] Wongwandee M, Linasmita P. Central nervous system melioidosis: A systematic review of individual participant data of case reports and case series. PLoS Negl Trop Dis. 2019;13:e0007320. 10.1371/journal.pntd.0007320 31022232 PMC6504113

[7] Sullivan RP, Marshall CS, Anstey NM, Ward L, Currie BJ. 2020 Review and revision of the 2015 Darwin melioidosis treatment guideline; paradigm drift not shift. PLoS Negl Trop Dis. 2020;14:e0008659. 10.1371/journal.pntd.0008659 32986699 PMC7544138

[8] Gora H, Hasan T, Smith S, Wilson I, Mayo M, Woerle C, et al. Melioidosis of the central nervous system; impact of the *bimABm* allele on patient presentation and outcome. Clin Infect Dis. 2022;•••:ciac111. 35137005 10.1093/cid/ciac111

[9] Mukhopadhyay C, Kaestli M, Vandana KE, Sushma K, Mayo M, Richardson L, et al. Molecular characterization of clinical *Burkholderia pseudomallei* isolates from India. Am J Trop Med Hyg. 2011;85:121–3. 10.4269/ajtmh.2011.11-0166 21734136 PMC3122355

[10] Limmathurotsakul D, Dance DA, Wuthiekanun V, Kaestli M, Mayo M, Warner J, et al. Systematic review and consensus guidelines for environmental sampling of *Burkholderia pseudomallei.* PLoS Negl Trop Dis. 2013;7:e2105. 10.1371/journal.pntd.0002105 23556010 PMC3605150

[11] Gee JE, Bower WA, Kunkel A, Petras J, Gettings J, Bye M, et al. Multistate outbreak of melioidosis associated with imported aromatherapy spray. N Engl J Med. 2022;386:861–8. 10.1056/NEJMoa2116130 35235727 PMC10243137

[12] Petras JK, Elrod MG, Ty M, Adams P, Zahner D, Adams A, et al. Notes from the field: *Burkholderia pseudomallei* detected in a raccoon carcass linked to a multistate aromatherapy-associated melioidosis outbreak—Texas, 2022. MMWR Morb Mortal Wkly Rep. 2022;71:1597–8. 10.15585/mmwr.mm7150a5 36520678 PMC9762901

[13] Matthews RJ, Smith S, Wilson I, Tjahjono R, Young S, Hanson J. Case report: vagal nerve neuritis associated with pulmonary melioidosis provides potential insights into the pathophysiology of neuromelioidosis. Am J Trop Med Hyg. 2023;108:1212–4. 10.4269/ajtmh.22-0694 37188337 PMC10540098

[14] St John JA, Walkden H, Nazareth L, Beagley KW, Ulett GC, Batzloff MR, et al. *Burkholderia pseudomallei* rapidly infects the brain stem and spinal cord via the trigeminal nerve after intranasal inoculation. Infect Immun. 2016;84:2681–8. 10.1128/IAI.00361-16 27382023 PMC4995904

[15] Walkden H, Delbaz A, Nazareth L, Batzloff M, Shelper T, Beacham IR, et al. *Burkholderia pseudomallei* invades the olfactory nerve and bulb after epithelial injury in mice and causes the formation of multinucleated giant glial cells in vitro. PLoS Negl Trop Dis. 2020;14:e0008017. 10.1371/journal.pntd.0008017 31978058 PMC7002012

[16] St John JA, Ekberg JA, Dando SJ, Meedeniya AC, Horton RE, Batzloff M, et al. *Burkholderia pseudomallei* penetrates the brain via destruction of the olfactory and trigeminal nerves: implications for the pathogenesis of neurological melioidosis. MBio. 2014;5:e00025. 10.1128/mBio.00025-14 24736221 PMC3993850

[17] Burnard D, Bauer MJ, Falconer C, Gassiep I, Norton RE, Paterson DL, et al. Clinical *Burkholderia pseudomallei* isolates from north Queensland carry diverse *bimABm* genes that are associated with central nervous system disease and are phylogenomically distinct from other Australian strains. PLoS Negl Trop Dis. 2022;16:e0009482. 10.1371/journal.pntd.0009482 35700198 PMC9236262

[18] Sarovich DS, Price EP, Webb JR, Ward LM, Voutsinos MY, Tuanyok A, et al. Variable virulence factors in *Burkholderia pseudomallei* (melioidosis) associated with human disease. PLoS One. 2014;9:e91682. 10.1371/journal.pone.0091682 24618705 PMC3950250

[19] Trevino SR, Klimko CP, Reed MC, Aponte-Cuadrado MJ, Hunter M, Shoe JL, et al. Disease progression in mice exposed to low-doses of aerosolized clinical isolates of *Burkholderia pseudomallei.* PLoS One. 2018;13:e0208277. 10.1371/journal.pone.0208277 30500862 PMC6267979

[20] Guyton AC. Measurement of the respiratory volumes of laboratory animals. Am J Physiol. 1947;150:70–7. 10.1152/ajplegacy.1947.150.1.70 20252828

[21] Marchetti R, Dillon MJ, Burtnick MN, Hubbard MA, Kenfack MT, Blériot Y, et al. *Burkholderia pseudomallei* capsular polysaccharide recognition by a monoclonal antibody reveals key details toward a biodefense vaccine and diagnostics against melioidosis. ACS Chem Biol. 2015;10:2295–302. 10.1021/acschembio.5b00502 26198038

[22] Stefan CP, Arnold CE, Shoemaker CJ, Zumbrun EE, Altamura LA, Douglas CE, et al. Transcriptomic analysis reveals host miRNAs correlated with immune gene dysregulation during fatal disease progression in the Ebola virus cynomolgus macaque disease model. Microorganisms. 2021;9:665. 10.3390/microorganisms9030665 33806942 PMC8005181

[23] Perkins JR, Dawes JM, McMahon SB, Bennett DL, Orengo C, Kohl M. ReadqPCR and NormqPCR: R packages for the reading, quality checking and normalisation of RT-qPCR quantification cycle (Cq) data. BMC Genomics. 2012;13:296. 10.1186/1471-2164-13-296 22748112 PMC3443438

[24] Reckseidler SL, DeShazer D, Sokol PA, Woods DE. Detection of bacterial virulence genes by subtractive hybridization: identification of capsular polysaccharide of *Burkholderia pseudomallei* as a major virulence determinant. Infect Immun. 2001;69:34–44. 10.1128/IAI.69.1.34-44.2001 11119486 PMC97852

[25] DeShazer D, Brett PJ, Woods DE. The type II O-antigenic polysaccharide moiety of *Burkholderia pseudomallei* lipopolysaccharide is required for serum resistance and virulence. Mol Microbiol. 1998;30:1081–100. 10.1046/j.1365-2958.1998.01139.x 9988483

[26] Burtnick MN, Brett PJ, Harding SV, Ngugi SA, Ribot WJ, Chantratita N, et al. The cluster 1 type VI secretion system is a major virulence determinant in *Burkholderia pseudomallei.* Infect Immun. 2011;79:1512–25. 10.1128/IAI.01218-10 21300775 PMC3067527

[27] Holden MT, Titball RW, Peacock SJ, Cerdeño-Tárraga AM, Atkins T, Crossman LC, et al. Genomic plasticity of the causative agent of melioidosis, *Burkholderia pseudomallei.* Proc Natl Acad Sci U S A. 2004;101:14240–5. 10.1073/pnas.0403302101 15377794 PMC521101

[28] Benanti EL, Nguyen CM, Welch MD. Virulent *Burkholderia* species mimic host actin polymerases to drive actin-based motility. Cell. 2015;161:348–60. 10.1016/j.cell.2015.02.044 25860613 PMC4393530

[29] Morris JL, Fane A, Sarovich DS, Price EP, Rush CM, Govan BL, et al. Increased neurotropic threat from *Burkholderia pseudomallei* strains with a *B. mallei*–like variation in the *bimA* motility gene, Australia. Emerg Infect Dis. 2017;23:740–9. 10.3201/eid2305.151417 28418830 PMC5403032

[30] Sitthidet C, Stevens JM, Chantratita N, Currie BJ, Peacock SJ, Korbsrisate S, et al. Prevalence and sequence diversity of a factor required for actin-based motility in natural populations of *Burkholderia* species. J Clin Microbiol. 2008;46:2418–22. 10.1128/JCM.00368-08 18495853 PMC2446894

[31] Jayasinghearachchi HS, Corea EM, Jayaratne KI, Fonseka RA, Muthugama TA, Masakorala J, et al. Biogeography and genetic diversity of clinical isolates of *Burkholderia pseudomallei* in Sri Lanka. PLoS Negl Trop Dis. 2021;15:e0009917. 10.1371/journal.pntd.0009917 34851950 PMC8824316

[32] Janesomboon S, Muangsombut V, Srinon V, Meethai C, Tharinjaroen CS, Amornchai P, et al. Detection and differentiation of *Burkholderia* species with pathogenic potential in environmental soil samples. PLoS One. 2021;16:e0245175. 10.1371/journal.pone.0245175 33411797 PMC7790303

[33] Welkos SL, Klimko CP, Kern SJ, Bearss JJ, Bozue JA, Bernhards RC, et al. Characterization of *Burkholderia pseudomallei* strains using a murine intraperitoneal infection model and in vitro macrophage assays. PLoS One. 2015;10:e0124667. 10.1371/journal.pone.0124667 25909629 PMC4409376

[34] Limmathurotsakul D, Funnell SG, Torres AG, Morici LA, Brett PJ, Dunachie S, et al.; Steering Group on Melioidosis Vaccine Development. Consensus on the development of vaccines against naturally acquired melioidosis. Emerg Infect Dis. 2015;21:e141480. 10.3201/eid2106.141480 25992835 PMC4451926

[35] Conejero L, Patel N, de Reynal M, Oberdorf S, Prior J, Felgner PL, et al. Low-dose exposure of C57BL/6 mice to *burkholderia pseudomallei* mimics chronic human melioidosis. Am J Pathol. 2011;179:270–80. 10.1016/j.ajpath.2011.03.031 21703409 PMC3123849

[36] Nelson M, Barnes KB, Davies CH, Cote CK, Meinig JM, Biryukov SS, et al. The BALB/c mouse model for the evaluation of therapies to treat infections with aerosolized *Burkholderia pseudomallei.* Antibiotics (Basel). 2023;12:506. 10.3390/antibiotics12030506 36978372 PMC10044689

[37] Bearss JJ, Hunter M, Dankmeyer JL, Fritts KA, Klimko CP, Weaver CH, et al. Characterization of pathogenesis of and immune response to *Burkholderia pseudomallei* K96243 using both inhalational and intraperitoneal infection models in BALB/c and C57BL/6 mice. PLoS One. 2017;12:e0172627. 10.1371/journal.pone.0172627 28235018 PMC5325312

[38] Stassart RM, Möbius W, Nave KA, Edgar JM. The axon-myelin unit in development and degenerative disease. Front Neurosci. 2018;12:467. 10.3389/fnins.2018.00467 30050403 PMC6050401

[39] Molina-Gonzalez I, Miron VE, Antel JP. Chronic oligodendrocyte injury in central nervous system pathologies. Commun Biol. 2022;5:1274. 10.1038/s42003-022-04248-1 36402839 PMC9675815

[40] Kenigsbuch M, Bost P, Halevi S, Chang Y, Chen S, Ma Q, et al. A shared disease-associated oligodendrocyte signature among multiple CNS pathologies. Nat Neurosci. 2022;25:876–86. 10.1038/s41593-022-01104-7 35760863 PMC9724210

[41] Graf LM, Rosenkranz SC, Hölzemer A, Hagel C, Goebell E, Jordan S, et al. Clinical presentation and disease course of 37 consecutive cases of progressive multifocal leukoencephalopathy (PML) at a German tertiary-care hospital: a retrospective observational study. Front Neurol. 2021;12:632535. 10.3389/fneur.2021.632535 33613439 PMC7890249

[42] Cortese I, Reich DS, Nath A. Progressive multifocal leukoencephalopathy and the spectrum of JC virus-related disease. Nat Rev Neurol. 2021;17:37–51. 10.1038/s41582-020-00427-y 33219338 PMC7678594

[43] Ramesh G, Borda JT, Dufour J, Kaushal D, Ramamoorthy R, Lackner AA, et al. Interaction of the Lyme disease spirochete *Borrelia burgdorferi* with brain parenchyma elicits inflammatory mediators from glial cells as well as glial and neuronal apoptosis. Am J Pathol. 2008;173:1415–27. 10.2353/ajpath.2008.080483 18832582 PMC2570132

[44] Trevino SR, Dankmeyer JL, Fetterer DP, Klimko CP, Raymond JLW, Moreau AM, et al. Comparative virulence of three different strains of *Burkholderia pseudomallei* in an aerosol non-human primate model. PLoS Negl Trop Dis. 2021;15:e0009125. 10.1371/journal.pntd.0009125 33571211 PMC7904162

[45] Taweechaisupapong S, Kaewpa C, Arunyanart C, Kanla P, Homchampa P, Sirisinha S, et al. Virulence of *Burkholderia pseudomallei* does not correlate with biofilm formation. Microb Pathog. 2005;39:77–85. 10.1016/j.micpath.2005.06.001 16084684

[46] Boisvert AA, Cheng MP, Sheppard DC, Nguyen D. Microbial biofilms in pulmonary and critical care diseases. Ann Am Thorac Soc. 2016;13:1615–23. 10.1513/AnnalsATS.201603-194FR 27348071 PMC5059503

[47] Chakraborty P, Bajeli S, Kaushal D, Radotra BD, Kumar A. Biofilm formation in the lung contributes to virulence and drug tolerance of *Mycobacterium tuberculosis.* Nat Commun. 2021;12:1606. 10.1038/s41467-021-21748-6 33707445 PMC7952908

[48] Nyanasegran PK, Nathan S, Firdaus-Raih M, Muhammad NAN, Ng CL. Biofilm signaling, composition and regulation in *Burkholderia pseudomallei.* J Microbiol Biotechnol. 2023;33:15–27. 10.4014/jmb.2207.07032 36451302 PMC9899790

[49] Petras JK, Elrod MG, Ty MC, Dawson P, O’Laughlin K, Gee JE, et al. Locally acquired melioidosis linked to environment—Mississippi, 2020–2023. N Engl J Med. 2023;389:2355–62. 10.1056/NEJMoa2306448 38118023 PMC10773590

