# Supplementary material for: Virulence of Burkholderia pseudomallei ATS2021 Unintentionally Imported to United States in Aromatherapy Spray
Source: Emerg Infect Dis. 2024 Oct;30(10):2056–69. doi: 10.3201/eid3010.240084 (PMC11431913; doi:10.3201/eid3010.240084)
Supplement: Appendix 2 — Additional information for study of virulence of Burkholderia pseudomallei ATS2021 unintentionally imported to united states in aromatherapy spray. [file 24-0084-Techapp-s2.pdf]

Article DOI: https://doi.org/10.3201/eid3010.240084

EID cannot ensure accessibility for supplementary materials supplied by authors. Readers who have difficulty accessing supplementary content should contact the authors for assistance.

Virulence of *Burkholderia pseudomallei* ATS2021 Unintentionally Imported to United States in Aromatherapy Spray

Appendix 2

Appendix 2 Table. Gene products of importance for oligodendrocyte function

Host Protein	Host Protein Function	References
Enpp6	This protein is a member of the nucleotide pyrophosphatase/phosphodiesterase family of enzymes that play an important role in regulating extracellular nucleotide metabolism. The encoded preproprotein undergoes proteolytic processing to generate a glycosylphosphatidylinositol (GPI)-anchored membrane protein that hydrolyzes choline-containing lysophospholipids such as glycerophosphocholine. It is frequently described as a marker of myelinating oligodendrocytes, and its protein expression is down-regulated in various neurodegenerative conditions including chronic traumatic encephalopathy (CTE) as well as normal aging, which are both implicated with impaired oligodendrocyte function. Mice lacking the encoded protein develop fatty liver and myelin sheath abnormalities. NCBI Gene: 320981	(*1*–*5*)
Gjb1 (Cx32)	This protein is a member of the gap junction protein family. The gap junction proteins are membrane-spanning proteins that assemble to form gap junction channels that facilitate the transfer of ions and small molecules between cells. Gjb1 is localized to oligodendrocytes and alterations in its expression and/or function have been implicated in numerous disorders of the central nervous system. According to sequence similarities at the nucleotide and amino acid levels, the gap junction proteins are divided into two categories, α and β. Mutations in this gene cause X-linked Charcot-Marie-Tooth disease, an inherited peripheral neuropathy. NCBI Gene: 2705	(*6*–*8*)
Opalin	This protein is predicted to be involved in regulation of oligodendrocyte differentiation. Opalin, a central nervous system-specific myelin protein, has been suggested to play a role in mammalian-specific myelin. NCBI Gene: 93377	(*9*,*10*)
Pllp	The plasmolipin protein is a main component of the myelin sheath and plays an important role in the development and normal function of the nervous system. It is known to be down-regulated in post-mortem examinations of brains from human patients experiencing Alzheimer's disease and major depressive disorders. Pllp has also been a suggested biomarker of schizophrenia and possibly Alzheimer's disease. NCBI Gene: 751090	(*11*–*14*)
Sox10	This protein is a member of the SOX (SRY-related HMG-box) family of transcription factors involved in the regulation of embryonic development. This protein acts as a nucleocytoplasmic shuttle protein and is important for neural crest and peripheral nervous system development. Mutations in this gene are associated with Waardenburg-Shah and Waardenburg-Hirschsprung disease. Sox10 is a key regulator in differentiation of peripheral glial cells. In mice that carry a spontaneous or a targeted mutation of Sox10, neuronal cells form in dorsal root ganglia, but Schwann cells or satellite cells are not generated. At later developmental stages, this lack of peripheral glial cells results in a severe degeneration of sensory and motor neurons. NCBI Gene: 6663	(*15*–*17*)
Ugt8a	The protein encoded by this gene belongs to the UDP-glycosyltransferase family. It catalyzes the transfer of galactose to ceramide, a key enzymatic step in the biosynthesis of galactocerebrosides, which are abundant sphingolipids of the myelin membrane of the central and peripheral nervous systems. Lower levels of Ugt8a expression have been observed in the LacQ140 transgenic mouse model, which is associated with changes in lipid biosynthesis and with it an array of myelin-related disorders. NCBI Gene: 7638	(*18*–*20*)

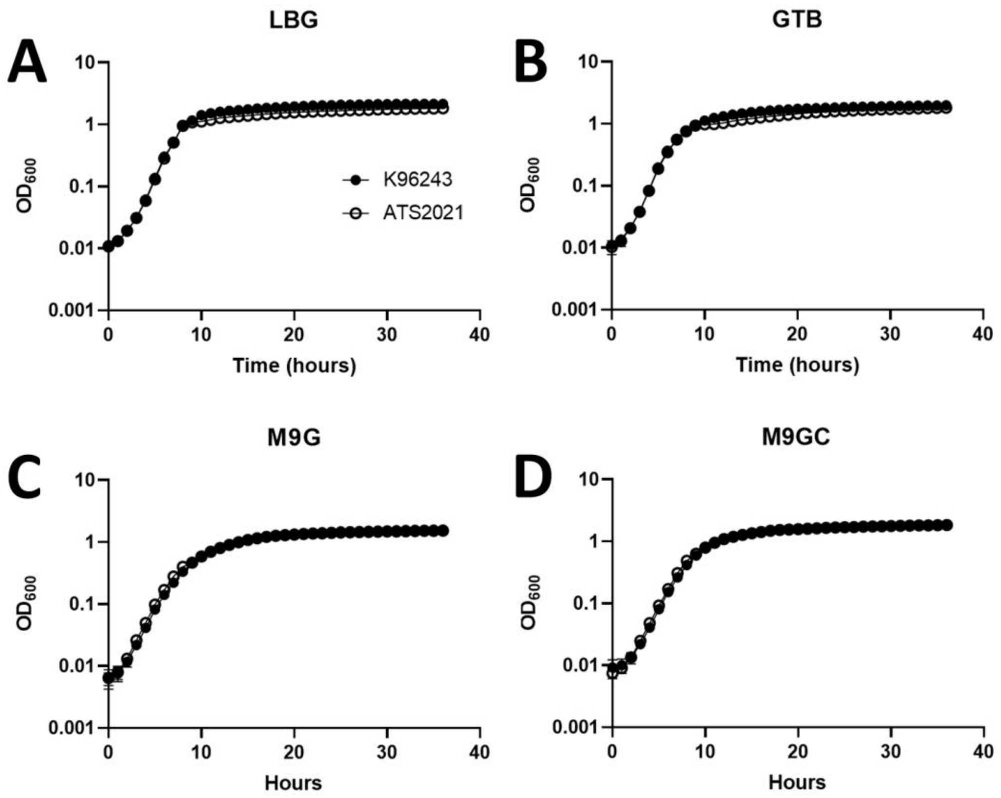

Appendix 2 Figure. Growth of K96243 and ATS2021 compared in several types of rich or defined media to assess differences with nutritional requirements.

References

1. Bi B, Choi HP, Hyeon SJ, Sun S, Su N, Liu Y, et al. Quantitative proteomic analysis reveals impaired axonal guidance signaling in human postmortem brain tissues of chronic traumatic encephalopathy. Exp Neurobiol. 2019;28:362–75. PubMed https://doi.org/10.5607/en.2019.28.3.362

2. Greiner-Tollersrud OK. The non-classical N-glycan processing pathway of bovine brain ecto-nucleotide phosphodiesterase/pyrophosphatase 6 (eNPP6) is brain specific and not due to mannose-6-phosphorylation. Neurochem Res. 2014;39:2025–9. PubMed https://doi.org/10.1007/s11064-014-1412-1

3. Sakagami H, Aoki J, Natori Y, Nishikawa K, Kakehi Y, Natori Y, et al. Biochemical and molecular characterization of a novel choline-specific glycerophosphodiester phosphodiesterase belonging to the nucleotide pyrophosphatase/phosphodiesterase family. J Biol Chem. 2005;280:23084–93. PubMed https://doi.org/10.1074/jbc.M413438200

4. Sziraki A, Lu Z, Lee J, Banyai G, Anderson S, Abdulraouf A, et al. A global view of aging and Alzheimer's pathogenesis-associated cell population dynamics and molecular signatures in human and mouse brains. Nat Genet. 2023;55:2104–16. PubMed https://doi.org/10.1038/s41588-023-01572-y

5. Zhang Y, Chen K, Sloan SA, Bennett ML, Scholze AR, O'Keeffe S, et al. An RNA-sequencing transcriptome and splicing database of glia, neurons, and vascular cells of the cerebral cortex. J Neurosci. 2014;34:11929–47. PubMed https://doi.org/10.1523/JNEUROSCI.1860-14.2014

6. Abrams CK, Goman M, Wong S, Scherer SS, Kleopa KA, Peinado A, et al. Loss of coupling distinguishes GJB1 mutations associated with CNS manifestations of CMT1X from those without CNS manifestations. Sci Rep. 2017;7:40166. PubMed https://doi.org/10.1038/srep40166

7. Bergoffen J, Scherer SS, Wang S, Scott MO, Bone LJ, Paul DL, et al. Connexin mutations in X-linked Charcot-Marie-Tooth disease. Science. 1993;262:2039–42. PubMed https://doi.org/10.1126/science.8266101

8. Kleopa KA, Abrams CK, Scherer SS. How do mutations in GJB1 cause X-linked Charcot-Marie-Tooth disease? Brain Res. 2012;1487:198–205. PubMed https://doi.org/10.1016/j.brainres.2012.03.068

9. Aruga J, Yoshikawa F, Nozaki Y, Sakaki Y, Toyoda A, Furuichi T. An oligodendrocyte enhancer in a phylogenetically conserved intron region of the mammalian myelin gene Opalin. J Neurochem. 2007;102:1533–47. PubMed https://doi.org/10.1111/j.1471-4159.2007.04583.x

10. Yoshikawa F, Sato Y, Tohyama K, Akagi T, Hashikawa T, Nagakura-Takagi Y, et al. Opalin, a transmembrane sialylglycoprotein located in the central nervous system myelin paranodal loop membrane. J Biol Chem. 2008;283:20830–40. PubMed https://doi.org/10.1074/jbc.M801314200

11. Fischer I, Cochary EF, Konola JT, Romano-Clark G. Expression of plasmolipin in oligodendrocytes. J Neurosci Res. 1991;28:81–9. PubMed https://doi.org/10.1002/jnr.490280108

12. Musunuri S, Wetterhall M, Ingelsson M, Lannfelt L, Artemenko K, Bergquist J, et al. Quantification of the brain proteome in Alzheimer's disease using multiplexed mass spectrometry. J Proteome Res. 2014;13:2056–68. PubMed https://doi.org/10.1021/pr401202d

13. Shulgin AA, Lebedev TD, Prassolov VS, Spirin PV. Plasmolipin and its role in cell processes. Mol Biol. 2021;55:773–85. PubMed https://doi.org/10.1134/S0026893321050113

14. Zhou B, Zhu Z, Ransom BR, Tong X. Oligodendrocyte lineage cells and depression. Mol Psychiatry. 2021;26:103–17. PubMed https://doi.org/10.1038/s41380-020-00930-0

15. Britsch S, Goerich DE, Riethmacher D, Peirano RI, Rossner M, Nave KA, et al. The transcription factor Sox10 is a key regulator of peripheral glial development. Genes Dev. 2001;15:66–78. PubMed https://doi.org/10.1101/gad.186601

16. Szeto IYY, Chu DKH, Chen P, Chu KC, Au TYK, Leung KKH, et al. SOX9 and SOX10 control fluid homeostasis in the inner ear for hearing through independent and cooperative mechanisms. Proc Natl Acad Sci U S A. 2022;119:e2122121119. PubMed https://doi.org/10.1073/pnas.2122121119

17. Turnescu T, Arter J, Reiprich S, Tamm ER, Waisman A, Wegner M. Sox8 and Sox10 jointly maintain myelin gene expression in oligodendrocytes. Glia. 2018;66:279–94. PubMed https://doi.org/10.1002/glia.23242

18. Dupree JL, Coetzee T, Blight A, Suzuki K, Popko B. Myelin galactolipids are essential for proper node of Ranvier formation in the CNS. J Neurosci. 1998;18:1642–9. PubMed https://doi.org/10.1523/JNEUROSCI.18-05-01642.1998

19. Shing K, Sapp E, Boudi A, Liu S, Seeley C, Marchionini D, et al. Early whole-body mutant huntingtin lowering averts changes in proteins and lipids important for synapse function and white matter maintenance in the LacQ140 mouse model. bioRxiv. 2023 Jul 20. https://doi.org/10.1101/2023.01.26.525697

20. Tsugawa H, Ishihara T, Ogasa K, Iwanami S, Hori A, Takahashi M, et al. A lipidome landscape of aging in mice. Nat Aging. 2024;4:709–26. PubMed https://doi.org/10.1038/s43587-024-00610-6
